# The role of the Nrf2/GSH antioxidant system in cisplatin resistance in malignant rhabdoid tumours

**DOI:** 10.1007/s00432-023-04734-x

**Published:** 2023-04-20

**Authors:** Patricia Hannon Barroeta, Maureen J. O’Sullivan, Daniela M. Zisterer

**Affiliations:** 1 School of Biochemistry and Immunology, Trinity Biomedical Sciences Institute, Trinity College Dublin, Pearse St, Dublin, D02 R590 Ireland; 2 The National Children’s Research Centre, Children’s Health Ireland at Crumlin, Dublin, D12 N512 Ireland

**Keywords:** Malignant rhabdoid tumour, Apoptosis, Chemoresistance, Nuclear erythroid-related factor-2, Glutathione, Reactive oxygen species

## Abstract

**Purpose:**

Malignant rhabdoid tumour (MRT) is a rare and aggressive childhood malignancy that occurs in the kidneys or central nervous system and is associated with very poor prognosis. Chemoresistance is a major issue in the treatment of this malignancy leading to an urgent need for a greater understanding of its underlying mechanisms in MRT and novel treatment strategies for MRT patients. The balance between oxidative stress mediated by reactive oxygen species (ROS) and the antioxidant system has become a subject of interest in cancer therapy research. Studies have implicated key players of the antioxidant system in chemotherapeutic including the well-known antioxidant glutathione (GSH) and the transcription factor nuclear erythroid-related factor-2 (Nrf2).

**Methods:**

This study evaluated the role of these components in the response of MRT cells to treatment with the commonly used chemotherapeutic agent, cisplatin.

**Results:**

This study characterised the basal levels of GSH, ROS and Nrf2 in a panel of MRT cell lines and found a correlation between the expression profile of the antioxidant defence system and cisplatin sensitivity. Results showed that treatment with ROS scavenger N-acetylcysteine (NAC) protected cells from cisplatin-induced ROS and apoptosis. Interestingly, depleting GSH levels with the inhibitor buthionine sulphoximine (BSO) enhanced cisplatin-induced ROS and sensitised cells to cisplatin. Lastly, targeting Nrf2 with the small molecule inhibitor ML385 or by siRNA diminished GSH levels, enhanced ROS and sensitised resistant MRT cells to cisplatin.

**Conclusions:**

These results suggest that targeting the Nrf2/GSH antioxidant system may present a novel therapeutic strategy to combat chemoresistance in rhabdoid tumours.

## Introduction

Malignant rhabdoid tumour (MRT) is a rare and highly metastatic paediatric neoplasm. These tumours typically arise in the kidneys and in the central nervous system where they are referred to malignant rhabdoid tumour of the kidney (MRTK) and as atypical teratoid rhabdoid tumours (ATRT), respectively. However, they can also occur in any other soft tissue sites (Geller et al. 2015). Due to their rare and aggressive nature, these tumours are often diagnosed at advanced stages and are associated with very poor clinical outcome (Geller et al. 2015; Xie et al. 2022; Tekautz et al. 2005). The platinum agent cisplatin is utilised in a number of treatment regimens for MRT but efficacy in the clinic is hampered by primary and acquired resistance. For this reason, further study into the molecular and cellular responses to cisplatin in MRT is warranted to gain a greater understanding of the mechanisms underlying cisplatin resistance.

Redox homeostasis is critical for cellular health and function. It is maintained through a balance between reactive oxygen species (ROS) and antioxidants. The cellular defence system against oxidative stressors, which are mediated by ROS and xenobiotic toxicants, utilise many antioxidants including vitamins, glutathione (GSH) generating enzymes (e.g. glutamate cysteine ligase) and phase 2 drug metabolising enzymes (e.g. glutathione S-transferases) (Ray et al. 2012). A disturbance in the balance between ROS and antioxidants has been associated with cancer progression and with chemoresistance (Liou and Storz 2010). Interestingly, elevated GSH levels have previously been linked with chemotherapeutic resistance including resistance to platinum-based anti-cancer agents (Bansal and Simon 2018). Levels of cellular thiols such as GSH play a critical role in detoxification of these platinum agents. GSH can bind and inactivate cisplatin through its reactive thiol group, preventing cisplatin from binding to DNA and inducing its damage (Silva et al. 2019). The expression of enzymes involved in GSH synthesis are regulated by the transcription factor Nrf2, a key regulator of the antioxidant response (Tonelli et al. 2018). Nrf2 activity is regulated by Keap1. Upon activation, Nrf2 dissociates from Kelch like ECH associated protein 1 (Keap1) and translocates to the nucleus where it binds to the antioxidant response element (ARE) and upregulates the transcription of a number of antioxidant genes such as glutathione s-transferase genes. Mutations in Keap1 and Nrf2 have been associated with poor survival in cancer patients (Traverso et al. 2013). Moreover, high Nrf2 expression has also been correlated with chemoresistant phenotype in several malignancies such as lung cancer and gallbladder cancer, as it can help cells overcome oxidative stress induced by anti-cancer drug treatment (Sporn and Liby 2012).

In the current study, we have investigated whether the Nrf2/GSH antioxidant system plays a role in determination of cisplatin cytotoxicity in a panel of MRT cells lines. More specifically, this study aimed to establish whether modulating the balance between ROS and the Nrf2/GSH antioxidant system may present a therapeutic strategy to sensitise MRT cells to cisplatin. This study reports elevated Nrf2 expression and cellular GSH levels, along with diminished basal ROS levels in a cisplatin-resistant MRT cell line, BT16, when compared to sensitive cells. Moreover, targeting ROS levels by treatment with a ROS scavenger significantly protected MRT cells from cisplatin-mediated apoptotic cell death. Depleting GSH levels with a GSH inhibitor enhanced cisplatin-induced ROS leading to an enhancement of cisplatin-mediated cell death. Lastly, targeting Nrf2, a key mediator of the antioxidant defence system, both through a pharmacological approach and through genetic knockdown sensitised MRT cells to cisplatin-induced cell death. These novel findings provide insights into the therapeutic potential of targeting components of the antioxidant system in MRT to circumvent chemoresistance.

## Materials and methods

### Reagents

Cisplatin (Sigma-Aldrich; cat no. A9165) was dissolved in sterile 0.9% NaCl. Antimycin A (Sigma-Aldrich; cat no. A8674) and ML385 (Sigma-Aldrich; cat no. SML1833) were dissolved in DMSO. N-acetylcysteine (Sigma-Aldrich; cat no. A9165) was prepared as a 100 mM stock in dH_2_O and the pH was adjusted as to 7. Buthionine sulphoximine (BSO) (Tocris; cat no. 6954) was prepared as a 20 mM stock solution in H_2_O.

Stock solutions were stored at − 20 °C and working solutions were prepared fresh in sterile PBS (Sigma-Aldrich; cat no. 14190–094) or dH_2_O the day of the treatment.

### Cell culture

The BT12 and BT16 cell lines were originally isolated from ATRT patients; a 2-month-old female patient and a 2-year-old-male, respectively. These cell lines were a generous donation from Peter Houghton, St. Jude Children's Research Hospital (Memphis, TN, USA). The G401 cell line was derived from a 3-month-old male MRTK patient. BT12 and BT16 cells were grown in Roswell Park Memorial Institute (RPMI) medium + GlutaMAX™ supplemented with 10% (v/v) Foetal Bovine Serum (FBS) and 1% (v/v) PenStrep (100 units/ml penicillin, 100 mg/ml streptomycin). G401 cells were a kind gift from Prof Maureen O’Sullivan in the National Children’s Research Centre and are available from ATCC. G401 cells were grown in Dulbecco’s Modified Eagles Medium (DMEM) + GlutaMAX™ supplemented with 10% (v/v) FBS and 1% (v/v) PenStrep (100units/ml penicillin, 100 mg/ml streptomycin). Cells were incubated at 37 °C with 5% CO_2_ (Coyle et al. 2019).

### Cell viability

BT12, BT16 and G401 cell lines were seeded in 96-well plates and left overnight to adhere. The following day, they treated with a range of concentrations of cisplatin for 24, 48 and 72 h. After the required incubation period, 20 μl of AlamarBlue™ (Invitrogen; Thermo Fisher Scientific, Inc. Waltham, MA, USA) was added to each well and the plate was left to incubate in the dark for 5 h at 37 °C. Fluorescence was measured on a SpectraMax plate reader at an excitation wavelength of 544 nm and an emission wavelength of 590 nm. All alamar blue assays were performed in triplicate. Dose–response curves were obtained and used to determine the IC50 values (GraphPad Prism 5.0).

### Apoptosis analysis

2 mL of cells were seeded in six well plates in complete media at seeding densities of 1 × 10^5^ cells/mL for BT12 and BT16 and 5 × 10^4^ cells/mL for G401 cells. The following day, the cells were treated with the relevant drugs for the required timepoint. Apoptosis analysis was performed through flow cytometric analysis of annexin V- fluorescein isothiocyanate (FITC, Accuscience; cat no. IQP–120F) and propidium iodide (PI, Sigma-Aldrich; cat no. P4170) stained cells as previously described (Coyle et al. 2019).

### Measurement of intracellular reactive oxygen species

Cells were seeded in 6 well plates at a density of 1 × 10^5^ cells/well in complete media and the following day were treated with the relevant drugs for the required time. After incubation, intracellular levels of ROS were measured by flow cytometric analysis of 2′,7′-dichlorodihydrofluorescein-diacetate (H_2_DCFDA, Sigma-Aldrich; cat no. D6883) stained cells as previously described (Magnano et al. 2021).

### Measurement of mitochondrial reactive oxygen species

Cells were seeded in six well plates at a density of 1 × 10^5^ cells/well in complete media and the following day were treated with the relevant drugs for the required time. After incubation, cells were harvested, washed with PBS then stained with MitoSOX™ Red (2.5 μM) (Thermofisher; cat no. M36008) for 15 min at 37 °C in the dark, and then washed with warm PBS and analysed by flow cytometry. The MFI values of Mitosox (510/580 nm) were obtained with the FL-2 channel using the bandpass filter of 585/340 nm on a BD Accuri C6 flow cytometer.

### Measurement of GSH and GSSG

The ratio of GSH (reduced) and GSSG (oxidised glutathione), an indicator of oxidative stress, was determined using the Promega GSH/GSSG-Glo™ kit (Promega; cat no. V6611). Cells were seeded in a 96 well plate and the following day they were treated with the relevant drugs for the required time. After treatment, media was removed, and the assay was conducted according to the manufacturer’s instructions and luminescence was measured using the Luminoskan™ Microplate Luminometer. The GSH/GSSG ratio was calculated using the formula: GSH/GSSG ratio = $\frac{\text{Total glutathion}-{GSSG}}{GSSG/2}$.

In this assay, two moles of GSH are generated from one mole of GSSG, a twofold adjustment was applied to the GSSG values to obtain an accurate measurement of the GSSG levels, as per the recommended guidelines.

### Western blot analysis

Western blotting of Nrf2 was conducted as previously described (Coyle et al. 2019). Blots were probed with an antibody from Cell Signalling Technology: Nrf2 (1:1000; cat no; 12,721). Blots were re-probed with anti-actin (1:10,000, Sigma-Aldrich, cat no. A3854) or anti-*γ*-tubulin (1:5000, Calbiochem, cat no. CP06) to ensure equal loading.

### Nrf2 knockdown using small interfering (si) RNA

The knockdown of Nrf2 was achieved using on-target plus human Nrf2 siRNA SMART pool (GE Healthcare Dharmacon, Inc.; cat no.), while non-targeting pool served as a negative control. Cells were seeded in six well plates at a density of 1 × 10^5^ cells/mL in antibiotic free media. The following day, Nrf2 siRNA or non-targeting siRNA was transfected into cells using Lipofectamine (Thermo Fisher; cat no. 11668–027) at a final concentration of 50 nM. Western blot analysis was employed to confirm Nrf2 knockdown at 24 h post-transfection.

### Statistical analysis

GraphPad Prism 5 (GraphPad Software, Inc., La Jolla, CA, USA) was used for statistical analysis of experimental data. Results are displayed as the mean ± standard error of the mean. Statistical analysis was performed using a one-way ANOVA with Tukey’s post hoc test or a paired t test for comparison of two groups. Values of *p < 0.05, **p < 0.01 and ***p < 0.001 were deemed statistically significant. Correlation analysis was performed using GraphPad Prism 5 by plotting percentage cell viability (measured by alamar blue) in response to cisplatin treatment (10 µM) against either relative GSH, relative Nrf2 expression or relative ROS levels. *R* values and *p* values were obtained from the correlation analysis.

## Results

### Susceptibility to cisplatin-mediated cells death is related to the antioxidant response

**Fig. 1:**
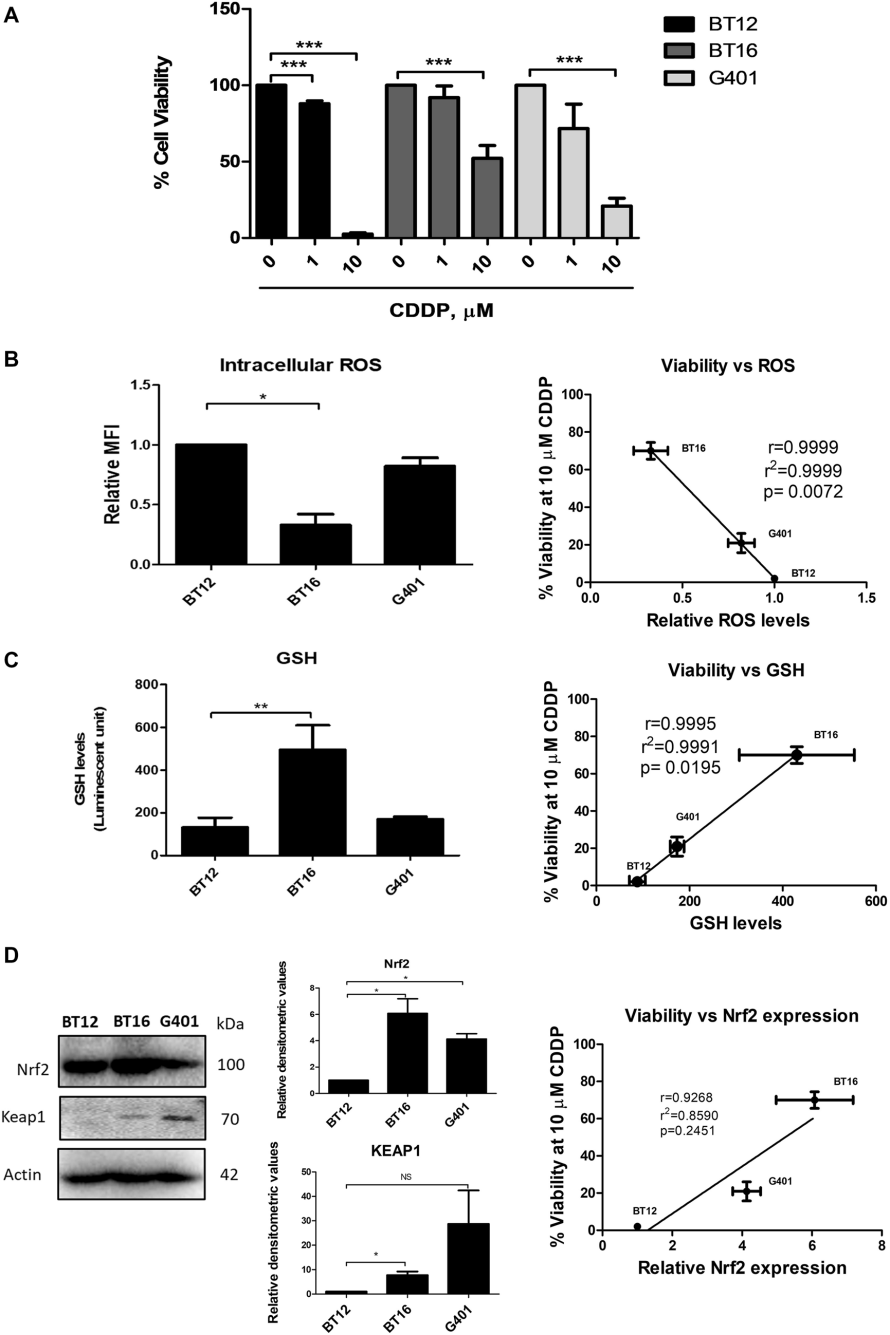
Cisplatin resistance in a panel of MRT cell lines correlates with the profile of ROS/GSH balance and Nrf2 expression. **A** Cells were treated with increasing concentrations of cisplatin (CDDP) or vehicle (0.009% NaCl) for 48 h. After this incubation period, cell viability was determined using the AlamarBlue™ viability assay. Values represent the mean ± S.E.M of three independent experiments performed in triplicate. **B** Basal ROS levels were assessed by flow cytometric analysis of H_2_DCFDA stained MRT cells and plotted against percentage viability of cells treated with 10 µM cisplatin for correlation analysis. Values represent the mean ± S.E.M of three independent experiments performed in triplicate.** C** Basal levels of GSH were assessed in a panel of MRT cells using the GSH/GSSG-Glo™ assay and measuring luminescence. The values are representative of mean ± S.E.M of five independent experiments. Correlation analysis was performed with values obtained from four independent experiments plotted against the percentage viability of MRT cells treated with 10 µM cisplatin and correlation was performed using GraphPad Prism. **D** Western blot analysis of basal expression of Nrf2 and Keap 1 and representative actin. The results are representative of mean ± S.E.M of three independent experiments. The values were plotted against percentage viability of MRT cells treated with 10 µM cisplatin and correlation analysis was performed using GraphPad Prism. Statistical analysis was performed using a paired *t* test. ***p < 0.001, **p < 0.001, *p < 0.05

Three MRT cell lines, BT12, BT16 and G401 were assessed to determine sensitivity to cisplatin, a commonly used chemotherapeutic agent for the treatment of MRT. The cells were incubated with 1 and 10 µM of cisplatin for 48 h, after which cell viability was measured. Previously, BT12 and BT16 cells have been used as a model for studying cisplatin resistance in MRT with BT12 cells designated as cisplatin-sensitive and BT16 cells designated as cisplatin-resistant (Coyle et al. 2019; Hannon Barroeta et al. 2022). In agreement with previous reports, the cell lines showed varying susceptibility to cisplatin treatment, with BT12 cells being the most sensitive, followed by G401 cells and BT16 being the most resistant (Fig. 1A). These results are in agreement with our previously published the IC50 values of 1.18 μM for BT12 cells, 5.13 μM for BT16 cells, and 1.45 μM for G401 cells (Hannon Barroeta et al. 2022).

To investigate the difference in sensitivities to cisplatin, the basal expression levels of three key components involved in redox homeostasis was assessed. ROS has been shown to be elevated in cancer cells due to their increased metabolism and hypoxic environment (Hayes et al. 2020). Moreover, high levels of basal intracellular ROS have been shown to confer cancer cells with vulnerability to ROS-inducing therapeutic agents such as cisplatin (Salatino et al. 2019; Zhu et al. 2018). Studies have demonstrated that cancer cells adapt to their high levels of ROS production by upregulating their antioxidant defence system which include levels of the primary antioxidant, glutathione and the transcription factor Nrf2 that regulates the expression of a wide array of antioxidant genes (No et al. 2014; Brozovic et al. 2010). Analysis of the intracellular levels of ROS showed that the cisplatin-sensitive BT12 cell line had significantly higher basal levels of intracellular ROS when compared to the cisplatin-resistant BT16 cells, with G401 cells having intermediary levels (Fig. 1B). Correlation analysis of ROS levels versus cell viability in response to 10 µM cisplatin was performed to visually present a potential correlation relationship between the two variables. A linear trend was observed suggesting inverse correlation (r value of 0.9999) between basal ROS levels and cisplatin resistance.

Glutathione is one of the key players involved in cisplatin detoxification as it is able to bind to this platinum agent preventing DNA damage. Basal levels of glutathione in the three cell lines were next examined, with the cisplatin-resistant BT16 cell lines having significantly higher levels than BT12 cells and G401 cells. This observation was consistent with the profile of basal ROS levels suggesting a relationship between the balance of glutathione, ROS and susceptibility to cisplatin in MRT. To further examine this relationship, the data were plotted in a graph of cell viability against relative basal glutathione levels and a linear relationship was observed with a statistically significant *r* value of 0.9995 confirming a correlation between glutathione and cisplatin resistance (Fig. 1C).

Lastly, the basal expression levels of Nrf2 and Keap1 were analysed using western blotting and BT16 was found to have significantly increased expression of both Nrf2 and Keap1 when compared to BT12 cells with intermediary levels of Nrf2 in G401 cells. As this trend was consistent with the aforementioned results of basal levels of GSH and ROS, a correlation analysis was also conducted to compare expression of Nrf2 and cellular viability in response to cisplatin treatment. Interestingly, G401 exhibited significantly higher levels of Nrf2 expression compared to BT12 cells, whereas this increase was not observed with GSH and ROS levels, where levels of GSH and ROS were comparable. This may be because the ROS/GSH balance is mediated not only by Nrf2, but other cellular components too that may vary between cells such as thioredoxin activity, glutathione peroxidase activity or NADPH generation (Liu et al. 2022). Some correlation was observed between Nrf2 and cisplatin resistance; however, this was not found to be statistically significant. This could be due to the low number of samples (3 cell lines) which is a limitation of the correlation analysis in this study. Nonetheless, this trend was consistent with the results obtained when looking at the basal profile of ROS and glutathione in relation to the sensitivity of the three MRT cell lines to cisplatin. These data suggest that resistance to cisplatin may be correlated to the antioxidant response.

### Cisplatin induces ROS production and oxidative stress in MRT cell lines

**Fig. 2:**
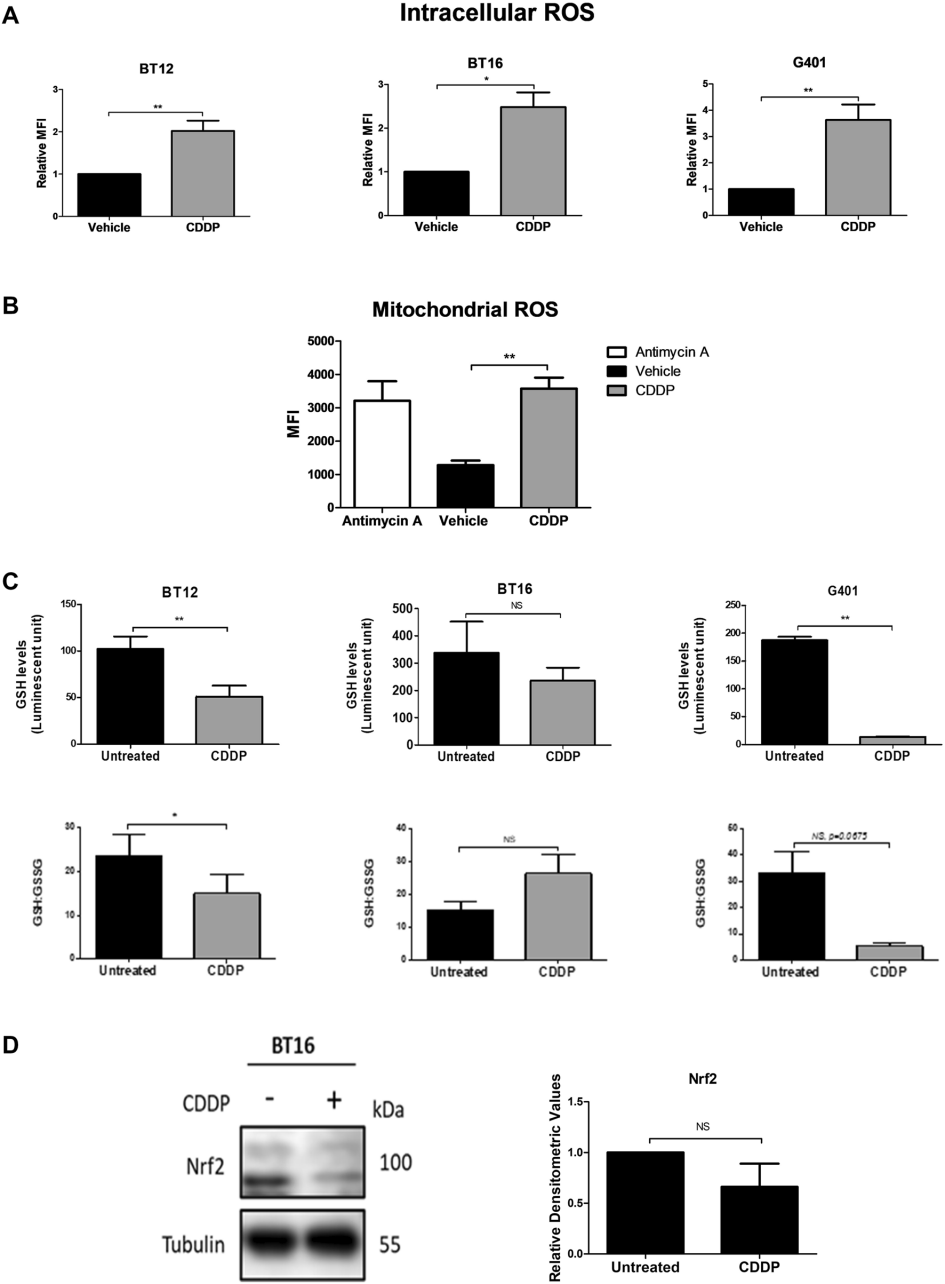
Cisplatin induces ROS and oxidative stress in a panel of MRT cells. **A** Cells were treated with cisplatin (CDDP) (1 µM for BT12 or 10 µM for BT16 and G401 cells) or vehicle (0.009% NaCl) for 48 h. ROS production was examined by flow cytometric analysis of H_2_DCFDA-stained cells. Values represent fold change relative to the vehicle control and are presented as the mean ± S.E.M of at least three independent experiments. **B** BT16 cells were treated with vehicle (0.009% NaCl), cisplatin (10 µM) or Antimycin A (10 µM for 1 h) as a positive control. Mitochondrial ROS levels were assessed by flow cytometric analysis of cells stained with MitoSOX™. Values represent the mean ± S.E.M of three independent experiments. **C** Levels of GSH and the ratio of GSH: GSSG in response to cisplatin treatment was assessed in BT12, BT16 and G401 cells. (BT12 cells were treated with 1 µM and BT16 and G401 cells were treated with 10 µM cisplatin respectively). **D** BT16 cells were treated with vehicle (0.009% NaCl) or treated with cisplatin (10 µM) for 48 h. Nrf2 expression was examined by western blot analysis. *γ*-Tubulin served as a loading control. Values were normalised by the loading control and are expressed as the fold change relative to the vehicle control. The results are representative of mean ± S.E.M of three independent experiments. Statistical analysis was performed using a paired *t*-test. **p < 0.001, *p < 0.05

As cisplatin resistance was found to correlate with high basal levels of glutathione and Nrf2 expression, as well as lower levels of intracellular ROS, this study next examined the effect of cisplatin on ROS, glutathione, GSH: GSSG ratio and Nrf2 levels. MRT cells were treated with cisplatin (1 µM for BT12 cells and 10 µM for BT16 and G401 cells due to their differential sensitivity e to cisplatin-induced apoptosis), and stained with H_2_DCFDA dye for flow cytometric analysis. The levels of intracellular ROS were significantly increased in response to cisplatin treatment in all three cell lines (Fig. 2A). To identify the source of ROS production in the cells, BT16 cells were also stained with Mitosox™ Red reagent. Antimycin A, which causes superoxide production from complex III of the mitochondrial electron transport chain was used as a positive control to measure mitochondrial ROS. A significant increase in fluorescence was observed in response to cisplatin treatment suggesting the source of intracellular ROS is the mitochondria (Fig. 2B). Next, the levels of GSH and the ratio of GSH to GSSG was examined as the ratio of reduced glutathione and oxidised glutathione is a marker of oxidative stress. Cisplatin treatment resulted in a significant reduction in GSH levels and an increase in oxidative stress in BT12 and G401 cells but notably, no significant effect was observed in resistant BT16 cells. Lastly, Nrf2 expression was examined in BT16 cells through western blot analysis (Fig. 2D. Western blot analysis depicted a double band. Densitometry was conducted on the lower band of 100 kDa. A trend of downregulation was observed in Nrf2 expression level. However, no significant change in Nrf2 was observed in response to cisplatin.

### Cisplatin-induced ROS mediates apoptosis and can be reversed by N-acetylcysteine

**Fig. 3:**
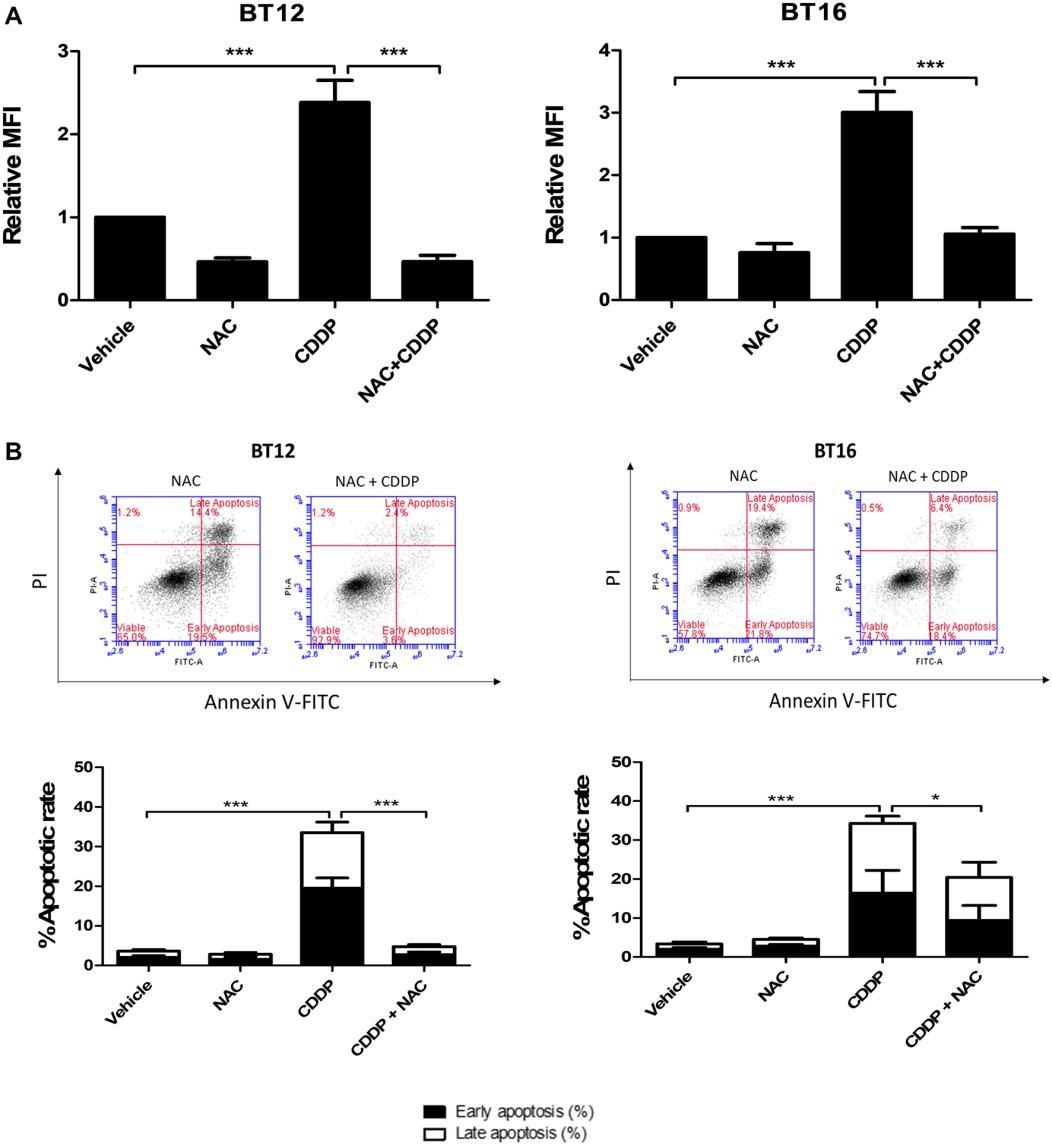
The antioxidant N-acetylcysteine abrogates cisplatin-induced ROS and apoptosis. BT12 and BT16 cells were pre-treated with NAC (5 mM) for 1 h before being treated with CDDP (1 µM and 10 µM, respectively) for 48 h. **A** The cells were stained with H_2_DCFDA for flow cytometric analysis. **B** The cells were stained with Annexin V/PI for flow cytometric analysis following a 48 h treatment with NAC, cisplatin, or a combination of both. Cells that were not stained with either Annexin V and PI were considered viable (quadrant 1). Cells that were stained with annexin V only and appeared in quadrant 2 were considered to be in early apoptosis. Cells that were positively stained for both annexin V and PI and appeared in quadrant 3, were considered to be in late apoptosis. The values represent mean ± S.E.M for three independent experiments. Statistical analysis was performed using a one-way ANOVA with Tukey’s post hoc test. ***p < 0.001, *p < 0.05

N-acetylcysteine (NAC) is a ROS scavenger and a synthetic precursor of cysteine and glutathione. It has a protective effect against ROS due to its free radical scavenging function by regulating the thiol redox status and increasing intracellular glutathione levels (Halasi et al. 2013). BT12 cells were treated with 1 μM cisplatin and BT16 cells were treated with 10 μM cisplatin due to their differential sensitivity to cisplatin and to induce a similar apoptotic rate. A significant increase in ROS was observed in response to cisplatin which was significantly reversed by NAC (Fig. 3A). In addition, pre-treatment with NAC also had a significant protective effect from apoptosis in both cisplatin-sensitive BT12 cells as well as cisplatin-resistant BT16 cells (Fig. 3B), indicating that ROS plays a key role in cisplatin-induced cytotoxicity and glutathione levels may modulate cisplatin sensitivity. Interestingly, NAC completely abrogated cisplatin-induced apoptosis in BT12 cells but only partially reduced cisplatin-induced apoptosis in BT16 cells. This may potentially be because the cell death occurring in BT12 cells in response to cisplatin is ROS-dependent whereas this may not totally be the case in BT16 cells, due to the demonstrated higher expression levels of cytoprotective components of the antioxidant system, GSH and Nrf2.

### Glutathione-mediated antioxidant defence modulates cisplatin sensitivity

**Fig. 4:**
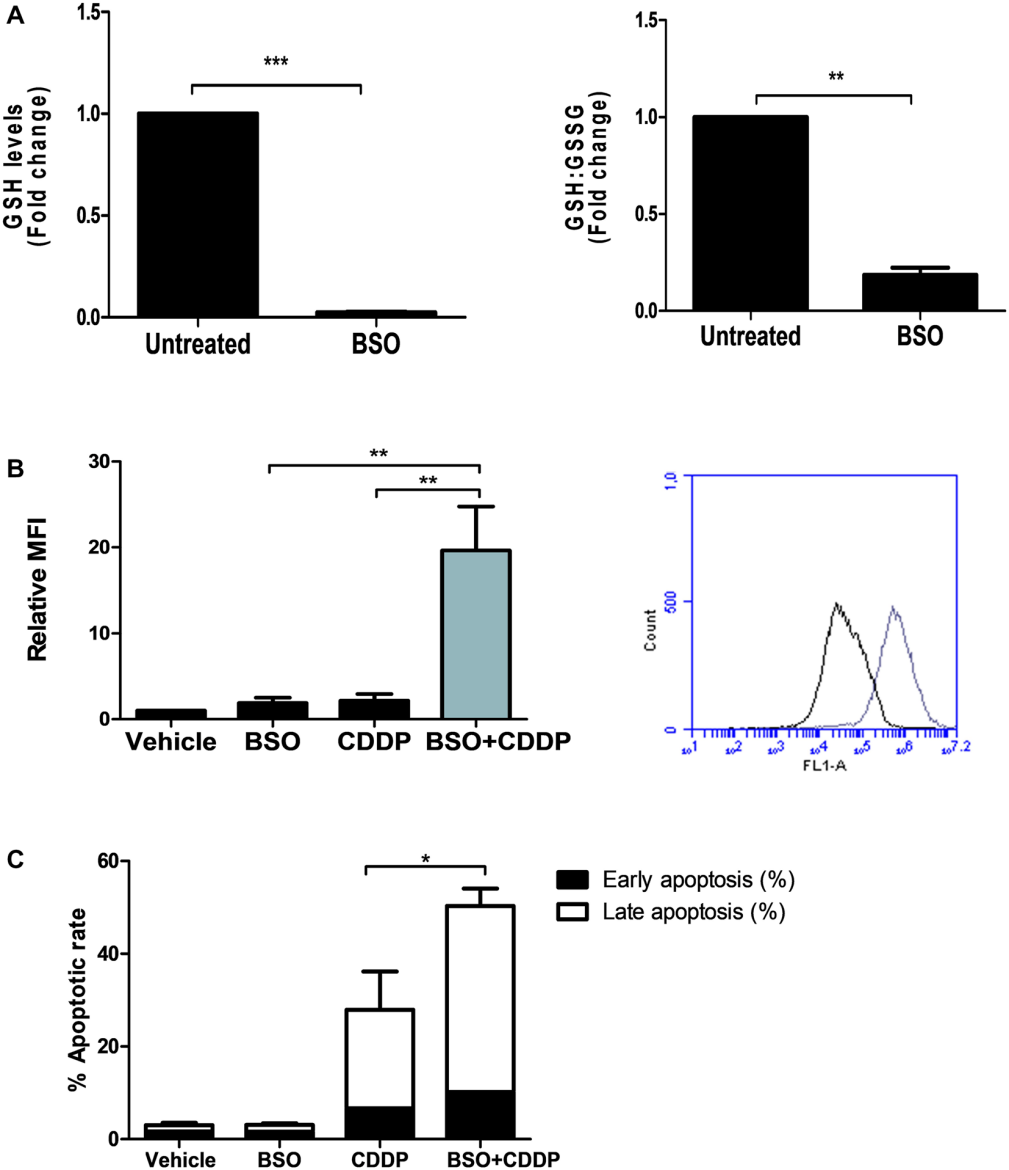
The GSH inhibitor BSO enhances cisplatin-induced ROS and sensitises resistant BT16 cells to cisplatin cytotoxicity. **A** BT16 cells were treated with BSO (200 µM) for 48 h. After this incubation period, GSH and the ratio of GSH:GSSG were examined. Values represent the mean ± S.E.M of three independent experiments and are expressed as the fold change relative to the vehicle control.** B** BT16 cells were treated with either BSO (200 µM) or cisplatin (CDDP, 10 µM) or a combination of both for 48 h. ROS levels were examined by flow cytometric analysis of H_2_DCFDA-stained cells. Values represent the mean ± S.E.M of three independent experiments and are expressed as the fold change relative to the vehicle control. Representative flow cytometric histogram depicts the shift in fluorescence between cisplatin-treated cells (black line) and cells treated with a combination of BSO and cisplatin (grey line) **C** BT16 cells were treated with either BSO (200 µM) or cisplatin (CDDP, 10 µM) or a combination of both for 48 h. Apoptosis was examined by flow cytometric analysis of Annexin V/PI-stained cells. Values represent the mean ± S.E.M of three independent experiments. Statistical analysis was performed using a paired *t*-test or a one-way ANOVA with Tukey’s post hoc test ***p < 0.001, **p < 0.001, *p < 0.05

Having observed that BT16 cells have high basal levels of glutathione and low basal levels of ROS, and that NAC treatment elicits a significantly protective effect against cisplatin, this study next evaluated the therapeutic potential of targeting glutathione in MRT. BT16 cells were treated with the glutathione inhibitor buthionine sulfoximine (BSO) which inhibits gamma-glutamylcysteine synthetase, the enzyme required in the first step of glutathione synthesis (Tagde et al. 2014). BSO treatment resulted in depletion of GSH levels and a significant decrease in GSH:GSSG ratio, confirming its inhibitory activity (Fig. 4A). Moreover, a statistically significant enhancement in ROS was observed in cells that were treated with BSO and cisplatin in combination compared to cells that were treated with BSO or cisplatin alone, approximately a 20-fold change in ROS levels (Fig. 4B). Cisplatin treatment alone induces approximately a 2.5 fold increase in ROS levels in BT16 cells (Fig. 2A) compared to vehicle To determine whether this increase in ROS production would impact the resistance of BT16 cells to cisplatin, cells were stained annexin V and PI and analysed. A significant increase in cisplatin induced apoptosis was observed (Fig. 4C), suggesting that modulating GSH and ROS levels may be a potential therapeutic strategy in MRT treatment.

### Nrf2 inhibition dampens the antioxidant response and sensitises cells to cisplatin

**Fig.5:**
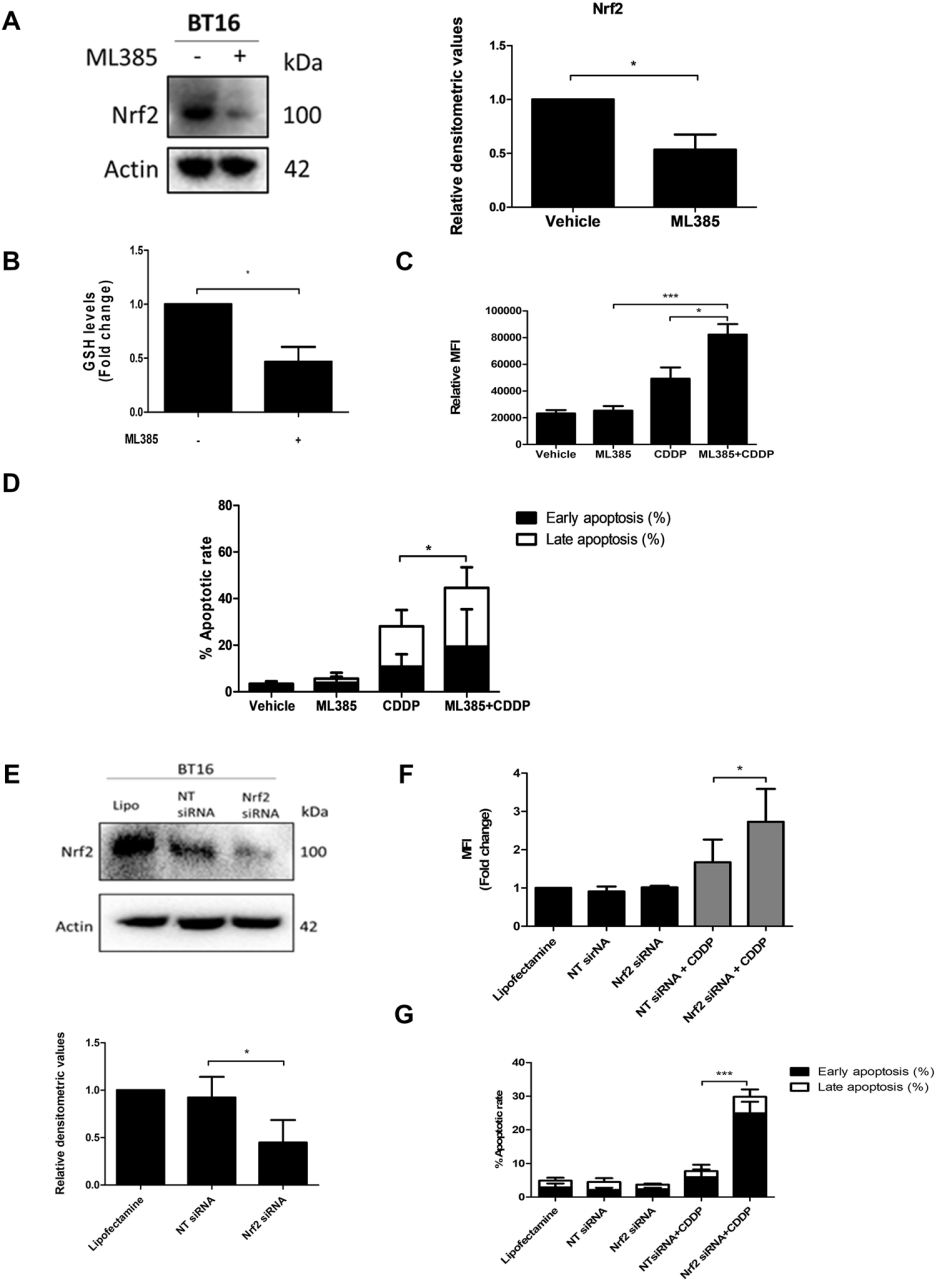
Inhibition of Nrf2 with the inhibitor ML385 and via genetic knockdown decreases GSH, upregulates ROS levels and sensitises cells to cisplatin-induced apoptosis. **A** BT16 cells were treated with ML385 (40 µM) for 48 h. Nrf2 expression was examined by western blot analysis. Actin served as a loading control. Values were normalised by the loading control and are expressed as the fold change relative to the vehicle control. **B** GSH levels following ML385 treatment. **C** BT16 cells were treated with either ML385 (40 µM) or cisplatin (10 µM) or a combination of both for 48 h. ROS levels were examined by flow cytometric analysis of H_2_DCFDA-stained cells. **D** BT16 cells were treated with either ML385 (40 µM) or cisplatin (10 µM) or a combination of both for 48 h. Apoptosis was examined by flow cytometric analysis of Annexin V/PI-stained cells. **E** BT1 6 were transfected with Non-Targeting (NT) control siRNA or with Nrf2 siRNA with Lipofectamine 2000™ (Lipo) for 24 h. Cells treated with Lipo alone served as negative control. Nrf2 levels were assessed by western blotting. Actin served as loading control. Densitometric analysis was performed using ImageLab. The results are representative of mean ± S.E.M of three independent experiments.** F** BT16 cells were transfected with NT and Nrf2 siRNA for 24 h alone or in combination with cisplatin (10 µM for 20 h). ROS levels were examined by flow cytometric analysis of H_2_DCFDA-stained cells** G** BT16 cells were transfected with NT and Nrf2 siRNA for 24 h alone or in combination with cisplatin (10 µM for 20 h). Values represent the mean ± S.E.M of three independent experiments. Statistical analysis was performed using a paired *t*-test or a one-way ANOVA with Tukey’s post hoc test. ***p < 0.001, *p < 0.05

Nrf2 is a master regulator of the antioxidant system. Resistant BT16 cells not only were observed to have higher basal levels of GSH, but also higher basal expression levels of Nrf2. Having observed that targeting GSH levels sensitised MRT cells to chemotherapeutic treatment, this study next evaluated the therapeutic potential of targeting Nrf2 in BT16 cells. To do this, the Nrf2 small molecule inhibitor ML385 was employed. Nrf2 was found to be significantly downregulated in response to ML385 (Fig. 5A) confirming its inhibitory activity. ML385 binds to Neh1, the Cap ‘N’ Collar Basic Leucine Zipper (CNC-bZIP) and downregulates total intracellular Nrf2 levels by inhibiting transcriptional activity of Nrf2 itself, as well as various downstream target genes. Moreover, a notable decrease in GSH was observed in response to ML385 treatment (Fig. 5B), confirming that GSH regulation is linked to Nrf2 activity. In addition, ML385 significantly enhanced both cisplatin-induced ROS and importantly, sensitised BT16 cells to cisplatin (Fig. 5C, D). To confirm this result, RNA interference was employed to silence Nrf2. As shown in Fig. 5E, Nrf2 expression was significantly decreased in BT16 cells transfected with Nrf2 siRNA when compared to cells transfected with NT siRNA. Moreover, a significant increase in ROS production and apoptosis was observed in cells transfected with Nrf2 siRNA and treated with cisplatin compared to cells that were transfected with NT siRNA and cisplatin (Fig. 5F, G). Collectively, these data suggest that high levels of GSH and Nrf2 expression may lead to chemoresistance in MRT and thus, may provide a novel therapeutic avenue in the treatment of MRT.

## Discussion

MRT is an aggressive paediatric tumour that can arise in young children and infants; often under the age of three. These tumours are associated with very poor survival and due to the early age of onset, treatment is linked to serious toxicities. Thus, there is an unmet medical urgency for novel therapeutic options to treat these tumours (Geller et al. 2015).

Cisplatin is a commonly utilised chemotherapeutic agent for the treatment of various cancers, including MRT. However, cisplatin treatment is associated with toxic side effects and tumour cells can develop resistance to its effects thereby limiting its efficacy (Florea and Büsselberg 2011). Many studies have examined the various proposed drug resistance mechanisms in cancers; one of which is the antioxidant defence system (Liu et al. 2016). Typically, cancer cells have higher intracellular ROS levels than normal cells due to their high metabolic rates. However, cells can upregulate their antioxidant system as a resistance mechanism in response to treatment with chemotherapeutic agents such as cisplatin to cope with drug-induced oxidative stress (Barrera 2021). This is primarily mediated by the transcription factor Nrf2, which is often referred to as a master regulator of the antioxidant response system and is involved in mediating the expression of a wide variety of antioxidant genes, including those involved in the GSH pathway (Suzuki et al. 2016). In the present study, we investigated whether the Nrf2/GSH pathway is a critical determinant in cytotoxicity mediated by cisplatin in MRT.

GSH is the most abundant antioxidant and is essential in maintaining cellular homeostasis. In chemo resistant cancer cells, elevated levels of GSH have been observed (Traverso et al. 2013). Low levels of GSH can lead to vulnerability to the cytotoxic effects of chemotherapeutic agents. In contrast, high levels of GSH can equip the cell with resistance to drug-induced oxidative stress (Ballatori et al. 2009). Indeed, the results presented in this study demonstrate that MRT cells with the highest resistance to cisplatin, the BT16 cells, also have the highest levels of basal GSH and Nrf2 expression as well as the lowest levels of intracellular ROS. BT12 cells, which were the most sensitive to cisplatin, were shown to have significantly higher levels of ROS, the lowest levels of GSH and the lowest basal expression of Nrf2. These results are in agreement with observations made by Neuwelt et al. who also demonstrated that BT16 cells have higher basal GSH levels compared to BT12 cells and also observed BT16 cells to be more resistant to cisplatin than BT12 cells (Neuwelt et al. 2014). Lastly, G401 cells appeared to have an intermediary phenotype in resistance, GSH levels, ROS levels and Nrf2 expression.

Cisplatin induces cell death in cancer cells by forming inter- and intra-strand DNA cross links inducing genotoxic stress, resulting in cytotoxicity. The cytotoxic effects of cisplatin are also associated with ROS production and oxidative stress. Marullo et al. have previously observed that independent to cisplatin-induced nuclear DNA damage, cisplatin cytotoxicity in non-small cell lung and prostate cancer cells is also linked to an upregulation in ROS production and oxidative stress that is mitochondrial dependent (Marullo et al. 2013). The present study assessed the effect of cisplatin on ROS, GSH and Nrf2 levels. ROS production was significantly elevated in the panel of MRT cell lines in response to cisplatin treatment. In order to determine whether this enhanced ROS production was coming from the mitochondria, BT16 cells were treated with cisplatin and stained with Mitosox™, a fluorogenic dye specifically targeted to the mitochondria that emits red fluorescence upon oxidation. A significant increase in fluorescence was observed in response to cisplatin treatment indicating that in agreement with previous reports in other cancer cells (Ballatori et al. 2009), cisplatin exposure induces a mitochondrial-dependent ROS response in MRT.

A decrease in GSH: GSSG ratio indicating increased oxidative stress was observed in BT12 and G401 cell lines. However, this increase in oxidative stress was not observed in BT16 cells, perhaps in part due the high basal GSH levels these cells exhibit. In agreement, increased inactivation of cisplatin by thiol-containing proteins has been reported to be an important mechanism underlying tumour resistance in many cancers including cervical cancer, osteosarcoma and glioblastoma (Silva et al. 2019; Zhu et al. 2016; Pasello et al. 2008; Rocha et al. 2016). Lastly, cisplatin treatment did not show marked effects on the overall level of expression of the transcription factor Nrf2.

To assess the role of ROS in cisplatin-induced toxicity on MRT cells, the cells were pre-treated with the ROS scavenger NAC prior to cisplatin treatment. NAC significantly reduced cisplatin-induced ROS generation and importantly abrogated the cytotoxic effects of cisplatin on both BT12 and BT16 cells indicating that cisplatin-induced apoptosis is mediated in part by ROS in MRT.

Having determined that ROS plays a major role in the cytotoxic effects of cisplatin and that GSH levels may correlate with chemoresistance, we next evaluated the potential of targeting GSH as a therapeutic strategy for MRT. In order to do this, the GSH inhibitor BSO was employed. BSO depletes GSH by inhibiting a critical enzyme in GSH synthesis, γ-glutamylcysteine synthase. BT16 cells were treated with 200 µM of the inhibitor for 48 h, a concentration which has no cytotoxic effects on the cells. GSH was depleted in response to BSO treatment, confirming the inhibitory effect of BSO in cells with high basal levels of GSH. Interestingly, a combination of BSO and cisplatin treatment resulted in a significant increase of ROS production and a significant enhancement in cisplatin-induced apoptosis. These results are in agreement with those published by Silva et al. who demonstrated that depletion of GSH in lung cancer cells with BSO resulted in a significant increase in sensitivity to cisplatin. Similarly, Li et al. have observed that sub-toxic concentrations of BSO synergistically enhanced the apoptotic response and the anti-proliferative activity of cisplatin and gemcitabine in biliary tract cancer cell lines (Li et al. 2016). Moreover, Neuwelt et al. also showed that combining cisplatin treatment with another glutathione inhibitor, acetaminophen, enhanced the effect of cisplatin (Neuwelt et al. 2014). This confirms the importance of GSH in determining cisplatin resistance and suggests that GSH could be a potential therapeutic target to overcome chemoresistance in MRT.

Based on these results, we hypothesised that levels of the transcription factor Nrf2 may determine cisplatin sensitivity in MRT cell lines by regulating GSH production. In order to test this hypothesis, a small molecule inhibitor of Nrf2, ML385, was employed to inhibit Nrf2 expression levels in our cisplatin-resistant BT16 cells. ML385 binds to the Cap ‘N’ Collar Basic Leucine Zipper (CNC-bZIP) domain of Nrf2 and interferes with the binding of Nrf2 to regulatory DNA-binding sequences thereby inhibiting downstream target gene expression. To further confirm the results obtained using pharmacological inhibition, a genetic approach to target Nrf2 using siRNA was also employed.

Inhibition of Nrf2 with ML385 resulted in a decrease of GSH and GSH:GSSG ratio in BT16 cells confirming that Nrf2 can regulate GSH production in MRT. Additionally, ML385 also enhanced cisplatin-induced ROS and cisplatin-mediated apoptotic cell death indicating the Nrf2/GSH antioxidant pathway modulates cisplatin resistance in MRT. Genetic inhibition of Nrf2 similarly showed a significant increase in cisplatin-induced ROS and importantly, a significant enhancement in cisplatin-induced apoptosis when comparing cells that were transfected with Nrf2 siRNA and treated with cisplatin to cells that were transfected with NT siRNA and treated with cisplatin.

Similar results were recently observed by Sun et al. who showed that ML385 enhanced ROS levels and cisplatin mediated cell death in ovarian cancer cells (Sun et al. 2019). Silva et al. also showed that knockdown of Nrf2 in lung cancer cells resulted in a significant decrease of GSH levels (Silva et al. 2019). Additionally, cells that were transduced with Nrf2 shRNA lentiviral recombinant vector were found to have higher sensitivity to cisplatin than their parental cell lines indicating Nrf2 mediated cisplatin resistance through GSH regulation. Moreover, Singh et al. reported that ML385 sensitised H460, a NSCLC tumour cell line, to a number of chemotherapeutic agents including carboplatin, paclitaxel and doxorubicin (Singh et al. 2016). Interestingly, approximately 40% of currently used anti-cancer drugs, including the anthracycline doxorubicin, have been reported to induce oxidative stress (Chen et al. 2007), indicating that targeting Nrf2 may be a useful strategy to overcome chemoresistance mediated by anti-cancer agents other than solely platinum based compounds.

In addition to alterations in the antioxidant system, it should be noted that Nrf2 inhibition may result in modulations in signal transduction pathways such as MAPK cascades. Furthermore, expression of multidrug resistance proteins, which exerts cisplatin efflux in cancer cells, has been shown to be regulated by Nrf2 (Bao et al. 2017). It would be of interest in future studies to examine the contribution of these components to alterations in cisplatin sensitivity following inhibition of the Nrf2 pathway in MRT.

In conclusion, this study has found a correlation between the expression profile of the antioxidant defence system and sensitivity to cisplatin in a panel of MRT cell lines. MRT cells with higher resistance to cisplatin exhibited higher levels of GSH and Nrf2 expression as well as lower levels of intracellular ROS. In response to cisplatin, ROS levels became significantly elevated in all cell lines examined and oxidative stress was increased in the two more cisplatin-sensitive cell lines. Administration of the ROS scavenger NAC elicited a protective effect from cisplatin-induced ROS and apoptosis indicating that ROS is an important mediator of cisplatin cytotoxicity in MRT. Most importantly, targeting either GSH with the inhibitor BSO or Nrf2 through an siRNA approach or with the small molecule inhibitor ML385 sensitised resistant MRT cells to cisplatin-induced cell death suggesting that targeting the Nrf2/GSH antioxidant defence pathway may present a novel therapeutic avenue in the design of new treatment strategies for MRT and a means of overcoming chemoresistance.

## Data Availability

The data generated and analysed in this article is included in this article. Materials used or analysed during the current study are available from the corresponding author on reasonable request.

## References

[CR1] Ballatori N et al (2009) Glutathione dysregulation and the etiology and progression of human diseases. Biol Chem 390:191–214 19166318 10.1515/BC.2009.033 PMC2756154

[CR2] Bansal A, Simon MC (2018) Glutathione metabolism in cancer progression and treatment resistance. J Cell Biol 217:2291–2298 29915025 10.1083/jcb.201804161 PMC6028537

[CR3] Bao L et al (2017) ABCF2, an Nrf2 target gene, contributes to cisplatin resistance in ovarian cancer cells. Mol Carcinog 56:1543–1553 28112439 10.1002/mc.22615 PMC5509336

[CR4] Barrera G et al (2021) Control of oxidative stress in cancer chemoresistance: spotlight on Nrf2 role. Antioxidants (basel) 10(4):510 33805928 10.3390/antiox10040510 PMC8064392

[CR5] Brozovic A, Ambriović-Ristov A, Osmak M (2010) The relationship between cisplatin-induced reactive oxygen species, glutathione, and BCL-2 and resistance to cisplatin. Crit Rev Toxicol 40:347–359 20163198 10.3109/10408441003601836

[CR6] Chen Y, Jungsuwadee P, Vore M, Butterfield DA, St Clair DK (2007) Collateral damage in cancer chemotherapy: oxidative stress in nontargeted tissues. Mol Interv 7:147–156 17609521 10.1124/mi.7.3.6

[CR7] Coyle R, Slattery K, Ennis L, Osullivan MJ, Zisterer DM (2019) The XIAP inhibitor embelin sensitises malignant rhabdoid tumour cells to TRAIL treatment via enhanced activation of the extrinsic apoptotic pathway. Int J Oncol 55:191–202 31115498 10.3892/ijo.2019.4804

[CR8] Florea AM, Büsselberg D (2011) Cisplatin as an anti-tumor drug: cellular mechanisms of activity, drug resistance and induced side effects. Cancer 3(1):1351–1371 10.3390/cancers3011351 PMC3756417 24212665

[CR9] Geller JI, Roth JJ, Biegel JA (2015) Biology and treatment of rhabdoid tumor. Crit Rev Oncog 20:199–216 26349416 10.1615/critrevoncog.2015013566 PMC6087667

[CR10] Halasi M et al (2013) ROS inhibitor N-acetyl-L-cysteine antagonizes the activity of proteasome inhibitors. Biochem J 454:201–208 23772801 10.1042/BJ20130282 PMC4322432

[CR11] Hannon Barroeta P, Magnano S, O’Sullivan MJ, Zisterer DM (2022) Evaluation of targeting autophagy for the treatment of malignant rhabdoid tumours. Cancer Treat Res Commun 32:100584 35679755 10.1016/j.ctarc.2022.100584

[CR12] Hayes JD, Dinkova-Kostova AT, Tew KD (2020) Oxidative stress in cancer. Cancer Cell 38:167–197 32649885 10.1016/j.ccell.2020.06.001 PMC7439808

[CR13] Li Q et al (2016) The effects of buthionine sulfoximine on the proliferation and apoptosis of biliary tract cancer cells induced by cisplatin and gemcitabine. Oncol Lett 11:474–480 26870236 10.3892/ol.2015.3879 PMC4727028

[CR14] Liou G-Y, Storz P (2010) Reactive oxygen species in cancer. Free Radic Res 44:479–496 20370557 10.3109/10715761003667554 PMC3880197

[CR15] Liu Y et al (2016) Cancer drug resistance: redox resetting renders a way. Oncotarget 7:42740–42761 27057637 10.18632/oncotarget.8600 PMC5173169

[CR16] Liu T, Sun L, Zhang Y, Wang Y, Zheng J (2022) Imbalanced GSH/ROS and sequential cell death. J Biochem Mol Toxicol 36:e22942 34725879 10.1002/jbt.22942

[CR17] Magnano S, Hannon Barroeta P, Duffy R, O’Sullivan J, Zisterer DM (2021) Cisplatin induces autophagy-associated apoptosis in human oral squamous cell carcinoma (OSCC) mediated in part through reactive oxygen species. Toxicol Appl Pharmacol 427:115646 34274415 10.1016/j.taap.2021.115646

[CR18] Marullo R et al (2013) Cisplatin induces a mitochondrial-ROS response that contributes to cytotoxicity depending on mitochondrial redox status and bioenergetic functions. PLoS ONE 8:e81162–e81162 24260552 10.1371/journal.pone.0081162 PMC3834214

[CR19] Neuwelt AJ et al (2014) Preclinical high-dose acetaminophen with N-acetylcysteine rescue enhances the efficacy of cisplatin chemotherapy in atypical teratoid rhabdoid tumors. Pediatr Blood Cancer 61:120–127 23956023 10.1002/pbc.24602 PMC5131918

[CR20] No JH, Kim YB, Song YS (2014) Targeting nrf2 signaling to combat chemoresistance. J Cancer Prev 19:111–117 25337579 10.15430/JCP.2014.19.2.111 PMC4204167

[CR21] Pasello M et al (2008) Overcoming glutathione S-transferase P1–related cisplatin resistance in osteosarcoma. Can Res 68:6661–6668 10.1158/0008-5472.CAN-07-5840 18701490

[CR22] Ray PD, Huang BW, Tsuji Y (2012) Reactive oxygen species (ROS) homeostasis and redox regulation in cellular signaling. Cell Signal 24:981–990 22286106 10.1016/j.cellsig.2012.01.008 PMC3454471

[CR23] Rocha CR, Kajitani GS, Quinet A, Fortunato RS, Menck CF (2016) NRF2 and glutathione are key resistance mediators to temozolomide in glioma and melanoma cells. Oncotarget 7:48081–48092 27344172 10.18632/oncotarget.10129 PMC5217002

[CR24] Salatino A et al (2019) H-Ferritin affects cisplatin-induced cytotoxicity in ovarian cancer cells through the modulation of ROS. Oxid Med Cell Longev 2019:3461251 31781333 10.1155/2019/3461251 PMC6875340

[CR25] Silva MM, Rocha CRR, Kinker GS, Pelegrini AL, Menck CFM (2019) The balance between NRF2/GSH antioxidant mediated pathway and DNA repair modulates cisplatin resistance in lung cancer cells. Sci Rep 9:17639 31776385 10.1038/s41598-019-54065-6 PMC6881285

[CR26] Singh A et al (2016) Small molecule inhibitor of NRF2 selectively intervenes therapeutic resistance in KEAP1-deficient NSCLC tumors. ACS Chem Biol 11:3214–3225 27552339 10.1021/acschembio.6b00651 PMC5367156

[CR27] Sporn MB, Liby KT (2012) NRF2 and cancer: the good, the bad and the importance of context. Nat Rev Cancer 12:564–571 22810811 10.1038/nrc3278 PMC3836441

[CR28] Sun X et al (2019) SIRT5 promotes cisplatin resistance in ovarian cancer by suppressing DNA damage in a ROS-dependent manner via regulation of the Nrf2/HO-1 pathway. Front Oncol 9:754 31456942 10.3389/fonc.2019.00754 PMC6700301

[CR29] Suzuki M, Otsuki A, Keleku-Lukwete N, Yamamoto M (2016) Overview of redox regulation by Keap1–Nrf2 system in toxicology and cancer. Curr Opin Toxicol 1:29–36

[CR30] Tagde A, Singh H, Kang MH, Reynolds CP (2014) The glutathione synthesis inhibitor buthionine sulfoximine synergistically enhanced melphalan activity against preclinical models of multiple myeloma. Blood Cancer J 4:e229 25036800 10.1038/bcj.2014.45 PMC4219442

[CR31] Tekautz TM et al (2005) Atypical teratoid/rhabdoid tumors (ATRT): improved survival in children 3 years of age and older with radiation therapy and high-dose alkylator-based chemotherapy. J Clin Oncol 23:1491–1499 15735125 10.1200/JCO.2005.05.187

[CR32] Tonelli C, Chio IIC, Tuveson DA (2018) Transcriptional regulation by Nrf2. Antioxid Redox Signal 29:1727–1745 28899199 10.1089/ars.2017.7342 PMC6208165

[CR33] Traverso N et al (2013) Role of glutathione in cancer progression and chemoresistance. Oxid Med Cell Longev 2013:972913 23766865 10.1155/2013/972913 PMC3673338

[CR34] Xie S et al (2022) Analysis on diagnosis and treatments of 16 cases of extracranial malignant rhabdoid tumor in children. Transl Cancer Res 11:629–638 35571638 10.21037/tcr-21-2548 PMC9091029

[CR35] Zhu H et al (2016) Molecular mechanisms of cisplatin resistance in cervical cancer. Drug Des Devel Ther 10:1885–1895 27354763 10.2147/DDDT.S106412 PMC4907638

[CR36] Zhu Z et al (2018) Glutathione reductase mediates drug resistance in glioblastoma cells by regulating redox homeostasis. J Neurochem 144:93–104 29105080 10.1111/jnc.14250

